# Synthesis, Antimicrobial Activity, and Molecular Docking Studies of Aminoguanidine Derivatives Containing an Acylhydrazone Moiety

**DOI:** 10.22037/ijpr.2020.113711.14446

**Published:** 2021

**Authors:** Xiaodong Yao, Hongmei Hu, Shiben Wang, Wenhao Zhao, Mingxia Song, Qiugui Zhou

**Affiliations:** a *Jiangxi Institute of Biological Products Inc, Ji’an , 343009, Jiangxi, China. *; b *Medical College, Jinggangshan University, Ji’an , 343009, Jiangxi, China. *; c *School of Pharmacy, Liaocheng University, LiaoCheng, 252059, Shandong, China. *; d *Research Center of Chinese Medicinal Resources and Functional Molecules of Jinggangshan University, Ji’an , 343009, China.*

**Keywords:** Aminoguanidine, Acylhydrazone, Antibacterial activity, Drug-resistance bacterial, Molecular docking

## Abstract

A series of aminoguanidine derivatives containing an acylhydrazone moiety was designed based on combination principles to find new antibacterial agents with wide spectra and high activities. The synthesized compounds were characterized by spectral methods and screened for their antibacterial activity. The results showed that several compounds provided great antimicrobial activities against Gram-positive bacteria (including the multidrug-resistant clinical isolates). Especially, this series of compounds presented high potency against *Staphylococcus aureus*, among which the derivative **3f** was the most promising one with a MIC value of 4 μg/mL. Compound** 3d,** with a tertiary butyl group, was found to have the broad spectrum inhibitory capacity, which is effective to eight strains and showed the most potent inhibitory activity against *B. subtilis* CMCC 63501 with a MIC value of 4 μg/mL. What’s more, compound **3d** also presented high activities against four multidrug-resistant strains, which were comparable or potent to oxacillin and penicillin. Molecular docking studies revealed that H-bond interaction with amino acid residue THR81 and alkyl hydrophobic interaction with residue ALA246 of FabH were crucial for their binding force and *in-vitro *antimicrobial activities.

## Introduction

With the increase of bacteria resistance, various drug-resistant bacteria are constantly being discovered. Methicillin-resistant *Staphylococcus aureus* (MRSA), vancomycin-resistant enterococci (VRE), multi-drug resistant *Escherichia coli*, and multi-drug resistant *Pseudomonas aeruginosa*, causing lethal diseases worldwide and great difficulties in the treatment of community-acquired and nosocomial infections (1-4), severely threatened global public health and resulted in high economic costs (5). A possible solution is to research and develop novel antibiotics with the new structure, target, and mechanism of action for the unmet needs to control the infections caused by resistant bacteria, which is always the core of attention for medicinal chemists (6).

**Figure 1:**
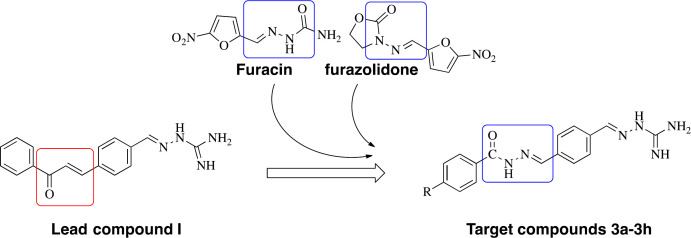
Lead compound and structure-based design of the target compounds

Aminoguanidine derivatives have recently got the attention of pharmaceutical chemists because of their diverse range of biological properties, including anticancer, antibacterial, antifungal, anti-inflammatory activities, and so on (7-10). In our previous work to find new antibacterial agents, several aminoguanidine derivatives were designed and synthesized, and their antibacterial potential was established (10-13). A series of aminoguanidine derivatives containing a chalcone moiety (Compound** I**, Figure 1) were found with potent antibacterial activity against Gram-positive strains, Gram-negative strains, and clinical isolates of multidrug-resistant (11). 

Acylhydrazones have also received considerable concerns because they possess a broad range of pharmacological properties, particularly antibacterial and anticancer activities (14-16). Acylhydrazone derivatives can easily form multiple hydrogen bonds with the proteins of microorganisms to increase the binding force of the receptor. Therefore, acylhydrazone compounds have been extensively researched to find new antimicrobial agents. Furacin and furazolidone, as representatives of clinical drugs containing the acylhydrazone moiety, play an important role in treating infections (17, 18). Recently, this scaffold was found to have important therapeutic targets on β-ketoacyl-acyl carrier protein synthase III (FabH) enzyme (19). FabH has an important role in the catalysis of branched-chain fatty acids, both in gram-positive and gram-negative bacteria. However, there are no significant homologous proteins in humans (20). 

Based on the above information, in this study, the structure-based design was employed to obtain novel molecules using compound **I **as the lead compound, in which the modification was focused on changing the unsaturated ketone to acylhydrazones with the expectation that the C=N group has a better binding with FabH. Thus, a series of new aminoguanidine derivatives containing an acylhydrazone moiety were synthesized, characterized, and screened for their antibacterial activity. Docking studies were carried out to determine the type of interactions between ligand and FabH, viz. hydrogen bonding and hydrophobic interactions.

## Experimental

*Instruments and Reagents*

 The reagents and solvents were purchased from Aladdin (Shanghai, China) or Sinopharm Chemical Reagent Co. Ltd. (Shanghai, China) and were used as received. Melting points were determined in open capillary tubes and are uncorrected. Reaction courses were monitored by thin-layer chromatography on silica gel-precoated F_254_ plates (Merck, Darmstadt, Germany). Developed plates were examined with UV lamps (254 nm). Nuclear magnetic resonance spectroscopy was performed on an AV-300 spectrometer (Bruker, Zurich, Switzerland) operating at 300 MHz for ^1^H and 75 MHz for ^13^C and using DMSO-*d*_6_ as solvent and tetramethylsilane as the internal standard. A MALDI‐TOF/ TOF mass spectrometer (Bruker Daltonik, Germany) was used to measure high-resolution mass spectroscopy. 

*General procedures for the synthesis of 4-substituent-benzohydrazides (* ***1a-1h*** *)*

Taking compound **1a** as an example: To a solution of benzoic acid (1.22 g, 10 mmol) in dry ethanol (10 mL), concentrated sulfuric acid (2 mL) was added, and the mixture was stirred at 90 ^o^C. After the completion of the reaction, 20% potassium carbonate solution was added into the mixture until no bubbles come out to remove the sulfuric acid and remained aromatic acid. The mixture was then extracted with dichloromethane (3 × 30 mL). The organic solution was dried over MgSO_4_, filtered and, concentrated under a vacuum to get the oily benzoates. 20 mL of hydrazine hydrate was added to the ethyl benzoates and refluxed at 120 ^o^C for 8 h. The solution was removed under vacuum to get the crude residue, which was recrystallized using EtOH to afford compounds **1a**. Yield 84%, White solid, m.p. 114-115 ºC, ^1^H-NMR (DMSO-*d*_6_, 300 MHz): *δ* 4.48 (s, 2H, NH_2_), 7.41-7.54 (m, 3H, Ph-H), 7.80-7.84 (m, 3H, Ph-H), 9.77 (s, 1H, CONH). ^13^C-NMR (DMSO-*d*_6_, 75 MHz): *δ* 127.39 , 128.76, 131.523, 133.76, 166.34. The compounds **1b-1h** were obtained using the same method. 

*General procedures for the synthesis of N’-(4-formylbenzylidene)-4-substituted-benzohydrazides (* ***2a-2h*** *)*

Taking compound **2a** as an example: To a solution of terephthalaldehyde (1.47 g, 11 mmol) in dry ethanol (10 mL) with 3 drops of AcOH, benzohydrazides（1.36 g, 10 mmol）was added in batches with the stirring at room temperature. After the completion of the reaction, the mixture was cooled. The precipitation was filtered and recrystallized by EtOH to give compound **2a**_._ Yield 61%, White solid, m.p. 198-200 ºC, ^1^H-NMR (DMSO-*d*_6_, 300 MHz): *δ* 7.52-7.62 (m, 3H, Ph-H), 7.92-8.01 (m, 6H, Ph-H), 8.54 (s, 1H, CH=N), 10.05 (s, 1H, CONH), 12.05 (s, 1H, CHO). ^13^C-NMR (DMSO-*d*_6_, 75 MHz): *δ *128.05, 128.17, 128.99, 130.44, 132.43, 133.67, 137.26, 140.32, 146.78, 163.78, 193.17. The compounds **2b-2h** were obtained using the same method. 

*General procedures for the synthesis of 2-(4-((2-(4-substituted benzoyl)hydrazineylid-ene)methyl)benzylidene)hydrazine-1-carboximidamide (* ***3a-3h*** *)*

Taking compound **3a** as an example: To a solution of aminoguanidine bicarbonate (0.67 g, 5 mmol) and compound **2a** (1.26 g, 5 mmol) in dry ethanol (20 mL), 4 drops of AcOH was added. And the mixture was stirred and refluxed for 8 h. The solvent was removed, and the resulted crude residue was applied onto a silica gel column eluted with 1%-2% CH_3_OH in CH_2_Cl_2 _to afford compound **3a**. The compounds **3b-3h** were obtained using the same method. The spectral data of compounds **3a-3h** were listed as below.

*(2-Benzoylhydrazono)methyl)benzylidene)hydrazine-1-carboximidamide* ***(3a)***

Yield 70.6%; white solid; m.p. 234-236 ºC. ^1^H-NMR (DMSO-*d*_6_, 300 MHz): *δ* 6.55 (s, 4H, Guanidine-H), 7.51-7.61 (m, 3H, Ph-H), 7.70 (d, 2H, *J* = 8.2 Hz, Ph-H), 7.81 (d, 2H, *J* = 8.2 Hz, Ph-H), 7.92 (d, 2H,* J* = 7.2 Hz, Ph-H), 8.05 (s, 1H, N=C-H), 8.45 (s, 1H, N=C-H), 11.89 (s, 1H, CONH). ^13^C-NMR (DMSO-*d*_6_, 75 MHz): *δ* 127.09, 127.27, 127.67, 128.52, 131.83, 133.40, 134.51, 143.46, 147.48, 158.73, 163.24, 175.81. ESI-HRMS calcd for C_16_H_17_N_6_O^+^ ([M + H]^+^): 309.1464; found: 309.1459.

*(2-(4-hydroxybenzoyl)hydrazono)methyl)benzylidene)hydrazine-1-carboximidamide* ***(3b)***

Yield 67.8%; white solid; m.p. 304-305 ºC,. ^1^H-NMR (DMSO-*d*_6_, 300 MHz): *δ* 6.51 (s, 4H, Guanidine-H), 6.86 (d, 2H, *J* = 8.4 Hz, Ph-H), 7.67 (d, 2H, *J* = 8.0 Hz, Ph-H), 7.76-7.82 (m, 4H, Ph-H), 8.03 (d, 1H, N=C-H), 8.41 (s, 1H, N=C-H), 11.65 (s, 1H, CONH). ^13^C-NMR (DMSO-*d*_6_, 75 MHz): *δ *115.08, 123.75, 127.10, 127.16, 129.74, 134.90, 136.69, 143.64, 146.40, 158.38, 160.87, 175.05. ESI-HRMS calcd for C_16_H_17_N_6_O_2_^+^ ([M + H]^+^): 325.1413; found: 325.1409.

*(2-(4-methylbenzoyl)hydrazono)methyl)benzylidene)hydrazine-1-carboximidamide* ***(3c)***

Yield 70.9%; white solid; m.p. 286-289 ºC. ^1^H-NMR (DMSO-*d*_6_, 300 MHz): *δ* 2.38 (s, 3H, CH_3_), 5.60 (s, 2H, Guanidine-H), 6.01 (s, 2H, Guanidine-H), 7.34 (d, 2H, *J* = 7.7 Hz, Ph-H), 7.66 (d, 2H, *J* = 8.1 Hz, Ph-H), 7.74 (d, 2H, *J* = 8.1 Hz, Ph-H), 7.83 (d, 2H, *J* = 7.7 Hz, Ph-H), 8.00 (s, 1H, N=C-H), 8.42 (s, 1H, N=C-H), 11.77 (s, 1H, CONH). ^13^C-NMR (DMSO-*d*_6_, 75 MHz): *δ *20.96, 126.41, 127.04, 127.56, 128.90, 130.59, 133.40, 138.61, 141.69, 142.43, 147.35, 160.75, 162.84. ESI-HRMS calcd for C_17_H_19_N_6_O^+^ ([M + H]^+^): 323.1620; found: 323.1615.

*(2-(4-(tert-butyl)benzoyl)hydrazono)methyl)benzylidene)hydrazine-1-carboximidamide* ***(3d)***

Yield 64.6%; white solid; m.p. 233-235 ºC. ^1^H-NMR (DMSO-*d*_6_, 300 MHz): *δ* 1.32 (s, 9H, C(CH_3_)_3_), 6.66 (s, 4H, Guanidine-H), 7.55 (d, 2H, *J* = 8.3 Hz, Ph-H), 7.70 (d, 2H, *J* = 8.2 Hz, Ph-H), 7.80 (d, 2H, *J* = 8.2 Hz, Ph-H), 7.87 (d, 2H, *J* = 8.3 Hz, Ph-H), 8.03 (d, 1H, N=C-H), 8.46 (d, 1H, N=C-H), 11.88 (s, 1H, CONH). ^13^C-NMR (DMSO-*d*_6_, 75 MHz): *δ* 30.99, 34.75, 125.26, 126.98, 127.20, 127.60, 130.75, 134.46, 137.40, 143.11, 147.33, 154.68, 159.25, 175.63. ESI-HRMS calcd for C_20_H_25_N_6_O^+^ ([M + H]^+^): 365.2090; found: 365.2078.

*(2-(4-methoxybenzoyl)hydrazono)methyl)benzylidene)hydrazine-1-carboximidamide* ***(3e)***

Yield 68.9%; white solid; m.p. 250-252 ºC. ^1^H-NMR (DMSO-*d*_6_, 300 MHz): *δ* 3.84 (s, 3H, OCH_3_), 6.84 (s, 4H, Guanidine-H), 7.06 (d, 2H, *J* = 7.5 Hz, Ph-H), 7.69 (d, 2H,* J* = 7.8 Hz, Ph-H), 7.80 (d, 2H,* J* = 7.8 Hz, Ph-H), 7.72 (d, 2H, *J* = 7.5 Hz, Ph-H), 8.04 (d, 1H, N=C-H), 8.45 (d, 1H, N=C-H), 11.80 (s, 1H, CONH). ^13^C-NMR (DMSO-*d*_6_, 75 MHz): *δ* 55.47, 113.72, 125.48, 127.11, 129.63, 134.79, 136.88, 143.46, 146.90, 158.67, 162.05, 162.69, 175.40. ESI-HRMS calcd for C_17_H_19_N_6_O_2_^+^ ([M + H]^+^): 339.1569; found: 339.1558.

*(2-([1,1’-biphenyl]-4-carbonyl)hydrazo-no)methyl)benzylidene)hydrazine-1-carboximidamide* ***(3f)***

Yield 63.5%; white solid; m.p. 337-340 ºC,. ^1^H-NMR (DMSO-*d*_6_, 300 MHz): *δ* 6.62 (s, 4H, Guanidine-H), 7.40-7.54 (m, 3H, Ph-H), 7.71-8.05 (m, 11H, Ph-H, N=C-H), 8.47 (s, 1H, N=C-H), 11.95 (s, 1H, CONH). ^13^C-NMR (DMSO-*d*_6_, 75 MHz): *δ* 126.72, 126.82, 126.97, 127.23, 128.21, 128.37, 129.11, 132.29, 134.02, 138.03, 139.17, 143.02, 147.61, 159.87, 162.78, 173.23. ESI-HRMS calcd for C_22_H_21_N_6_O^+^ ([M + H]^+^): 385.1777; found: 385.1766.

*(2-(4-chlorobenzoyl)hydrazono)methyl)benzylidene)hydrazine-1-carboximidamide* ***(3g)***

Yield 66.8%; white solid; m.p. 291-293 ºC. ^1^H-NMR (DMSO-*d*_6_, 300 MHz): *δ* 6.75 (s, 4H, Guanidine-H), 7.62 (d, 2H,* J* = 8.4 Hz, Ph-H), 7.72 (d, 2H,* J* = 8.4 Hz, Ph-H), 7.82 (d, 2H,* J* = 8.4 Hz, Ph-H), 7.96 (d, 2H,* J* = 8.4 Hz, Ph-H), 8.05 (s, 1H, N=C-H), 8.45 (s, 1H, N=C-H), 11.27 (s, 1H, CONH). ^13^C-NMR (DMSO-*d*_6_, 75 MHz): *δ* 127.22, 127.34, 128.65, 129.65, 132.11, 134.63, 136.97, 143.46, 147.76, 158.38, 162.19, 175.23. ESI-HRMS calcd for C_16_H_16_ClN_6_O^+^ ([M + H]^+^): 343.1074; found: 343.1068.

*(2-(4-bromobenzoyl)hydrazono)methyl)benzylidene)hydrazine-1-carboximidamide* ***(3h)***

Yield 71.1%; white solid; m.p. 324-325 ºC. ^1^H-NMR (DMSO-*d*_6_, 300 MHz): *δ *5.75 (s, 2H, Guanidine-H)), 6.09 (s, 2H, Guanidine-H)), 7.68 (d, 2H, *J* = 8.1 Hz, Ph-H), 7.76 (d, 4H, Ph-H), 7.88 (d, 2H, *J* = 8.1 Hz, Ph-H), 8.01 (s, 1H, N=C-H), 8.43 (s, 1H, N=C-H), 11.95 (s, 1H, CONH). ^13^C-NMR (DMSO-*d*_6_, 75 MHz): *δ* 125.53, 126.65, 127.28, 127.60, 129.79, 131.57, 133.56, 138.59, 142.71, 148.07, 160.46, 162.21. ESI-HRMS calcd for C_16_H_16_BrN_6_O^+^ ([M + H]^+^): 387.0569; found: 387.0562.

*Evaluation of Antibacterial Activity in-vitro*

 The antibacterial activity *in-vitro* against *S. aureus* (CMCC(B) 26003 and CMCC 25923,* S. pyogenes* CMCC 32067, *E. faecalis *CMCC 29212, *B. subtilis *CMCC 63501; *E. coli* CMCC 25922 and CMCC 44568, *P. aeruginosa *CMCC 27853 and CMCC 10104, as well as four methicillin-resistant clinical isolates (*S. aureus* ATCC 43300 and ATCC 33591, *E. coli* ATCC BAA-196, and *P. aeruginosa* ATCC BAA-2111), was evaluated using a two-fold serial dilution technique, and the final concentrations of compounds obtained were in the range of 0.5–128 μg/ml. Test bacteria were grown to mid-log phase in Mueller-Hinton broth (MHB) or Tryptone Soya Broth (TSB) and diluted 1000-fold in the same medium. The 10^5 ^CFU/ml bacteria were inoculated into MHB or TSB and dispensed at 0.2 ml/well in a 96-well microtiter plate. As positive controls, norfloxacin, oxacillin, and penicillin were used. Test compounds were prepared in DMSO, the final concentration of which did not exceed 0.05%. The MIC was defined as the concentration of a test compound that inhibited bacteria growth by more than 80% during 24 h incubation at 37 ^o^C. Bacteria growth was determined by measuring the absorption at 630 nm using a microtiter enzyme-linked immunosorbent assay (ELISA) reader. All tests were triple holes.

*Docking Studies*

Molecular docking studies were carried out for the synthesized compounds with the *E. coli *FabH-CoA complex structure (PDB ID: 1HNJ) using the Discovery Studio (version 2019) (19). The structures of compounds **3a-3h** were drawn using ChemBioDraw Ultra [Chemical Structure Drawing Standard; Cambridge Soft Corporation, USA (2010)], and then energetically minimized using Discovery Studio. The co-crystallized protein-ligand complex structure (pdb id: 1HNJ) was downloaded from Protein Data Bank and prepared as per the requirement of docking study, such as hydrogen atoms adding and water/impurities removing. The binding site was defined based on the volume occupied by the bound ligand in the “Define and Edit Binding site” tools of DS 2019. The input site sphere was built with a radius of 12 in x = 32.5723, y = 20.4034, and z = 29.1248. And other parameters remained at the default status. The compounds 3a-3h were docked with the receptor, and the LibDockScores were provided. Types of interactions of compound **3d **with the protein were analyzed after molecular docking.

*Prediction of ADME properties*

A computational study of titled compounds was performed for the prediction of ADME properties. Polar surface area (TPSA), miLog P, number of rotatable bonds (n-ROTB), number of hydrogen bond donor (HBD) and acceptor (HBA) atoms and violations of Lipinski’s rule of ﬁve were calculated using Molinspiration online property calculation toolkit (21,22). Absorption (%ABS) was calculated by: % ABS = 109 - (0.345*PSA) (23).

## Results and Discussion

*Chemistry*

**Scheme 1:**
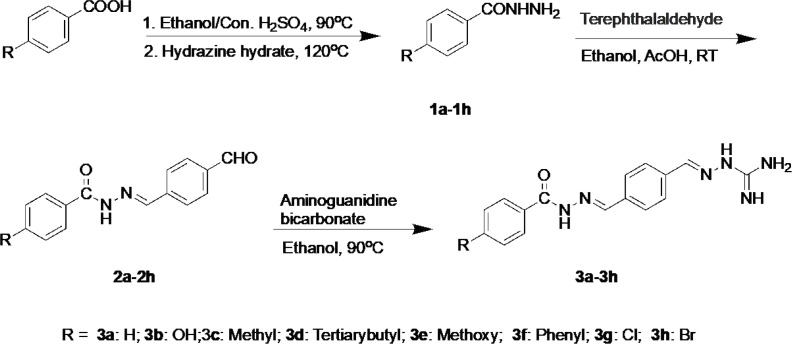
Synthetic scheme for the synthesis of compounds **3a-3h**

The synthetic route to prepare a new class of 2-(4-((2-(4- substituted benzoyl)hydrazineylidene)methyl)benzylidene)hydrazine-1-carboximidamides from 4-substituted benzoic acid is depicted in Scheme 1. The reaction of 4-substituted benzoic acid with the alcohol in the presence of concentrated sulfuric acid produced ethyl 4-substituted-benzoates under refluxing, which transformed into the corresponding benzohydrazides immediately (**1a-1h**). Compounds **2a-2h** were prepared by condensation of **1a-1h **with terephthalaldehyde in the presence of AcOH. Finally, the target compounds **3a-3h** were obtained by condensation of **2a-2h **with aminoguanidine bicarbonate. The structures of the target compounds were well characterized by ^1^H-NMR, ^13^C-NMR, and high-resolution mass spectrometry. 

Taking compound **3a** as an example in the structure confirmation. In the ^1^H-NMR spectrum, a single peak due to N-H of guanidyl was observed at 6.55 ppm. And the aromatic protons of the terminal benzene ring were observed in 7.51-7.61 and 7.92. A doublet of doublets (*J *= 8.2 Hz) due to aromatic protons of *para*-substituted phenyl ring was observed at 7.70 and 7.81 ppm. Two single peaks were found to absorb C-H in imine at 8.05 ppm or 8.45 ppm, respectively. A single peak due to N-H of amide was found at 11.89 ppm. The absorption peak in the hydrogen spectrum is completely in conformity with the hydrogen signal in the structure. The ^13^C NMR spectra also give accurate information about the structure of the compound, which involved 12 kinds of carbon in different chemical environments. Moreover, the high-resolution mass spectrometry of **3a** displayed an [M + H]^+^ signal at m/z 309.1459, which was corresponding to its molecular weight of 309.1464.

*Antimicrobial Activity*

 All of the target compounds (**3a-3h**) were evaluated for their *in vitro* antibacterial activity using a serial dilution method to obtain the minimum inhibitory concentration (MIC) against five gram-positive strains (*S. aureus* (CMCC(B) 26003 and CMCC 25923, *S. mutans* BNCC 336931, *E. faecalis* CMCC 29212 and *B. subtilis* CMCC 63501), and four gram-negative strains (*E. coli* CMCC 25922 and CMCC 44568 and *P. aeruginosa* CMCC 27853 and CMCC 10104) as well as four mutidrug-resistant clinical isolates (*S. aureus *ATCC 43300,* S. aureus *ATCC 33591,* E. coli *ATCC BAA-196, and *P. aeruginosa *ATCC BAA-2111). Norfloxacin, oxacillin, and penicillin were used as positive control drugs. 

The results of target compounds (**3a-3h**) were described in Table 1 as MIC values against the Gram-positive and Gram-negative strains. Generally compounds **3a-3h** presented the antibacterial activities but didn’t achieve the expected level, which were lower than that of the lead compound and positive controls. This may be caused by introducing too many nitrogen atoms in a molecule, which may result in too much polarity, and then result in low membrane permeability and low antibacterial activity. It could be found that some of the tested compounds showed potent to moderate inhibitory effects against the strains with MICs in 4-64 μg/mL. Compounds **3a**-**3c** hardly showed inhibitory activity at 64 μg/mL against the nine strains selected, the only compound **3b** exhibited moderate inhibition against *S. aureus* CMCC(B) 26003. Compound** 3d**, with a tertiary butyl group, is effective to eight strains and showed the most potent inhibitory activity against *B. subtilis* CMCC 63501 with a MIC value of 4 μg/mL. Compound **3e**, bearing a methoxy group，presented weaker activity only effective against* S. aureus* (CMCC(B) 26003, *B. subtilis* CMCC 63501 and* P. aeruginosa* CMCC 10104 with a MIC value of 64 μg/mL. Compound **3f**, bearing a phenyl group, presented better activity against* S. aureus* (CMCC(B) 26003, *B. subtilis* CMCC 63501, and* P. aeruginosa* CMCC 10104 with a MIC value of 4 μg/mL. Compound** 3g **and** 3h**, with a chlorine and bromine atom, respectively, are effective to seven strains with MICs value of 16, 32, or 64 μg/mL.

All the compounds were also subjected to evaluate the antibacterial activity against four multidrug-resistant clinical isolates (*S. aureus *ATCC 43300,* S. aureus *ATCC 33591,* E. coli *ATCC BAA-196, and *P. aeruginosa *ATCC BAA-2111). As shown in Table 2. compound **3d **also presented high activities (MIC = 8 μg/mL) against four multidrug-resistant strains, which was comparable or potent to oxacillin (MIC = 64 or 8 μg/mL) and penicillin (MIC ≥ 32 μg/mL). 

*Molecular docking*

**Figure 2:**
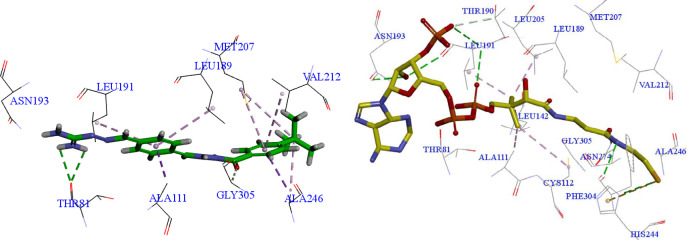
Interactions of compound** 3d **(left) and co-crystallized ligand** MLC **(right) with *E. coli *FabH

FabH receptor (also called *β*-ketoacyl-acyl carrier protein synthase III receptor) is a condensing enzyme that plays key role in fatty acid biosynthesis (24). It has been an important target for novel antibacterial drug design (25, 26). To illustrate the probable binding pattern, molecular docking between the aminoguanidine derivatives (**3a-3h**) and FabH receptor (PDB ID: 1HNJ) was performed (19, 27). Among them, tertbutyl derivative **3d** showed the strongest binding affinity with a binding score of 116.62, according to the antibacterial activity results. However, the co-crystallized ligand MLC exhibited a binding score of 147.50, which was much higher than that of the synthesized compounds. It suggested that these compounds might be weak in the binding with FabH, thereby showed antibacterial activity not good enough. The docking of compound** 3d** and FabH receptor was analyzed to provide the detailed binding interactions (Figure 2). The C=O and NH_2_ groups of compound **3d** function as an H-bond acceptor and donor, respectively, involved in two H-bonds formation with THR81 and GLY305. The THR81 as one of the crucial residues for the catalytic activity of FabH in various bacteria has been reported by Zhang *et al.* in their previous study (19). The two phenyl groups in **3d** were responsible for forming Pi hydrophobic interaction and hydrophobic force interaction with LEU189, LEU191, MET207, ALA246, ALA111, VAL212. It is worth mentioning that the critical amino acid residues MET207 and ALA246 also formed alkyl hydrophobic interaction with the tertbutyl group of **3d**, which explains the best antibacterial activity of **3d** in the previous screening. The importance of residue ALA246 in the binding with FabH receptor was also confirmed by Muhammad’s study, in which CYS112 and ALA246 were found to be responsible for substrate binding and cleavage of the alkyl chain of CoA (28). The co-crystallized ligand** MLC **showed more H-bonds interactions with FabH than **3d**, including the H-bonds formations withHIS 244, ASN274, PHE204, LEU191, ASN193. 

*Prediction of ADME properties*

A computational study was conducted to predict the ADME properties and drug-likeness of all of the synthesized compounds (Table 4). It has been demonstrated experimentally that the intestinal absorption of drugs is significantly correlated with their polar surface area (PSA). The PSA of a molecule effectively represents the portion of its surface belonging to polar atoms, such as oxygen, nitrogen, and attached hydrogens, and is a descriptor related to the passive molecular transport of a molecule through membranes. With this in mind, PSA could be used to predict the transport properties of drugs in the intestines (29). Palm *et al.* (30) have proven, based on Caco-2 cell studies, that drugs with a PSA value below 60 were completely absorbed in the intestine. For the target compounds **3a-h**, the PSA values ranged from 115.70 to 135.96 Å^2^ and the level of intestinal absorption (%ABS), according to the algorithm described by Zhao *et al.* (23), it was in the range of 62.1 to 69.1%. These findings indicated that these compounds would possess weak transport properties in the intestines.

The “Rule of 5” was established as a set of simple molecular descriptors by Lipinski based on the observation that most drugs are relatively small and lipophilic molecules (21). This rule states that most “drug-like” molecules have common parameters, including LogP ≤ 5, Mw ≤ 500, number of hydrogen bond acceptors ≤ 10, and number of hydrogen bond donors ≤ 5. Molecules violating more than one of these rules may have problems with bioavailability. 

**Table 1:** Inhibitory activity (MIC, μg/mL) of compounds **5a-5h **against Gram-positive and Gram-negative bacteria

**Compd.**	**R**	**Gram-positive strains**					**Gram-negative strains**			
		**26003** ^a^	**25923** ^b^	**29212** ^c^	**63501** ^d^	**336931** ^e^	**25922** ^f^	**44568** ^g^	**27853** ^h^	**10104** ^i^
**3a **	H	>64	>64	>64	>64	>64	>64	>64	>64	>64
**3b**	OH	**32**	>64	>64	64	>64	>64	>64	>64	>64
**3c**	CH_3_	>64	>64	>64	>64	>64	>64	>64	>64	>64
**3d**	C(CH_3_)_3_	**8**	**8**	**>64**	**4**	**32**	**8**	**16**	**16**	**8**
**3e**	OCH_3_	64	>64	>64	64	>64	>64	64	>64	64
**3f**	C_6_H_5_	4	>64	>64	4	>64	32	>64	>64	4
**3g**	Cl	32	32	>64	16	32	64	64	>64	32
**3h**	Br	32	64	>64	16	>64	16	32	64	32
Norfloxacin		0.125	0.125	1	2	16	0.125	0.125	2	4
Oxacillin		0.125	0.125	128	>128	0.125	128	>128	>128	128
Penicillin		0.125	0.125	128	128	0.125	128	>128	>128	32

^a^*Staphylococcus aureus *CMCC(B)26003; ^b^*Staphylococcus aureus* CMCC 25923; ^c^*Enterococcus faecalis *CMCC 29212; ^d^*Bacillus subtilis* CMCC 63501;^ e^*Streptococcus mutans *BNCC 336931;^ f^*Escherichia coli* CMCC 25922; ^g^*Escherichia coli* CMCC 44568; ^h^*pseudomonas aeruginosa* CMCC 27853;^ i ^*pseudomonas aeruginosa* CMCC 10104.

**Table 2:** Inhibitory activity (MIC, µg/mL) of compounds **5a-5h** against clinical isolates of multidrug-resistant strains

**Compd.**	**multidrug-resistant Gram-positive strains**		**multidrug-resistant Gram-negative strains**	
	**43300** ^a^	**3359** ^b^	**BAA-196** ^c^	**BAA-2111** ^d^
**3a **	>32	>32	>32	>32
**3b**	>32	>32	>32	>32
**3c**	>32	>32	>32	>32
**3d**	**8**	**8**	**8**	**8**
**3e**	>32	>32	>32	>32
**3f**	>32	32	32	16
**3g**	32	32	32	>32
**3h**	16	16	32	32
Norfloxacin	0.5	0.25	0.5	1
Oxacillin	64	8	ND	ND
Penicillin	32	>32	ND	ND

^a^*Staphylococcus aureus* ATCC 43300; ^b^*Staphylococcus aureus *ATCC 33591; ^c^multi-drug resistant *Escherichia coli *ATCC BAA-196; ^d^multi-drug resistant* Pseudomonas aeruginosa* ATCC BAA-2111; sND: not detected.

**Table 3:** Docking scores of compounds 3a-3h and co-crystallized ligand MLC in the docking with *E. coli* FabH

**Compounds**	**LibDockScore**
3a	107.269
3b	109.01
3c	107.32
3d	116.52
3e	111.048
3f	113.32
3g	107.55
3h	109.008
MLC	147.50

**Table 4:** Pharmacokinetic parameters which are important for good oral bioavailability and drug-likeness of target compounds **5a-p**

**Compds**	**ABS (%)**	**TPSA (Å** ^2^ **)**	**n-ROTB**	**MW**	**miLogP**	HBD	HBA	**Lipinski’s violation**
rule	-	-	≤10	<500	≤5	≤5	≤10	≤1
**3a**	69.1	115.73	6	308.4	2.3	5	7	0
**3b**	62.1	135.96	6	324.3	1.82	6	8	1
**3c**	69.1	115.70	6	322.4	2.75	5	7	0
**3d**	69.1	115.70	7	302.4	4.01	5	7	0
**3e**	65.9	124.96	7	338.4	2.36	5	8	0
**3f**	69.1	115.73	7	384.4	4.10	5	7	0
**3g**	69.1	115.73	6	342.8	2.98	5	7	0
**3h**	69.1	115.73	6	387.2	3.11	5	7	0

## Conclusion

For the first time, we synthesized a series of novel aminoguanidine derivatives containing an acylhydrazone moiety and determined their antibacterial activities against Gram-positive and Gram-negative bacteria. Some compounds had potential antibacterial activities against Gram-positive bacteria (including multidrug-resistant strains of clinical isolates). In particular, compound** 3d** with a tertiary butyl group was found to have the broad spectrum inhibitory capacity, which is effective to eight strains and showed the most potent inhibitory activity against *B. subtilis* CMCC 63501 with a MIC value of 4 μg/mL. Compound **3d **also presented high activities against four multidrug-resistant strains comparable to or potent than oxacillin and penicillin. Molecular docking studies revealed that H-bond interaction with amino acid residue THR81 and alkyl hydrophobic interaction with residue LA246 of FabH found to be crucial for their binding force and in vitro antimicrobial activities. What’s more, considering the performance of the antibacterial activities and the prediction of ADME properties, the acylhydrazone and aminoguanidine moieties are unsuited to coexist to avoid the low cell permeability.
